# Leukemic Retinopathy: A Diagnostic Clue for Initial Detection and Prognosis of Leukemia

**DOI:** 10.7759/cureus.50587

**Published:** 2023-12-15

**Authors:** Tatyana Beketova, Emanuel Mordechaev, Brian Murillo, Max D Schlesinger

**Affiliations:** 1 Ophthalmology, New York Medical College, Valhalla, USA; 2 Ophthalmology/Retina, Westchester Medical Center/New York Medical College, Valhalla, USA

**Keywords:** leukemia, retinopathy, eosinophilic leukemia, chronic eosinophilic leukemia, leukemic retinopathy

## Abstract

Leukemia is a systemic malignancy that can compromise various physiological functions, including vision. We report a case of a 37-year-old male presenting with worsening bilateral central vision loss, fatigue, shortness of breath, and ankle edema. Ophthalmic examination revealed extensive retinal hemorrhages, Roth spots, and subhyaloid hemorrhages, consistent with leukemic retinopathy. Further hematologic workup confirmed chronic eosinophilic leukemia. The patient showed systemic and visual improvement after prompt treatment with imatinib. This case highlights the importance of ophthalmological assessment in diagnosing leukemia, as ocular manifestations may often be the first sign of hematological disease.

## Introduction

Leukemia is a malignancy of the bone marrow that arises from the abnormal proliferation and differentiation of hematopoietic stem cells. This results in the accumulation of immature or abnormal blood cells in the marrow and peripheral blood [1]. Ocular manifestations of leukemia can impair vision. Of all leukemic ocular involvements, leukemic retinopathy is the most common, occurring in up to 50% of patients [2-4]. Further classification divides leukemic retinopathy into primary and secondary retinopathy. Primary retinopathy is characterized by direct retinal infiltration of cancerous leukocytes [5,6]. Secondary retinopathy is a sequela of leukemic hematological abnormalities, including thrombocytopenia, anemia, and hyperviscosity [5].

## Case presentation

A 37-year-old Hispanic male presented with a two-day history of progressively worsening central vision in both eyes. The patient’s past medical history was significant only for diet-controlled hyperlipidemia. There was no past ocular history and no relevant family history. The patient worked as a forklift operator and denied alcohol and illicit drug use. Upon review of systems, the patient revealed that he had been having fatigue, shortness of breath on exertion, and bilateral ankle swelling for two weeks. He denied fever, chills, and recent weight loss.

**Figure 1:**
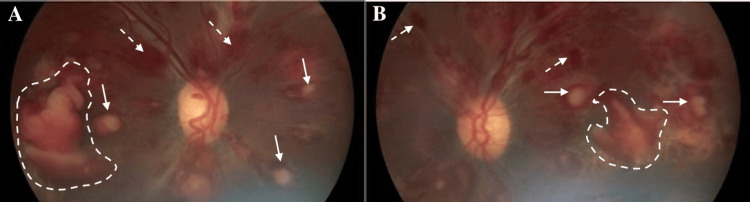
Color fundus photographs showing evidence of leukemic retinopathy Color fundus photography of patient’s right (A) and left (B) eyes at initial presentation depicting Roth spots (solid white arrows), as well as extensive intraretinal (dashed white arrows) and chronic subhyaloid hemorrhages (white outlines).

The best corrected visual acuity (BCVA) was counting fingers at 3 feet bilaterally. Pupils, intraocular pressure, confrontational visual fields, and motility were within normal limits bilaterally. Ishihara color plates were 0/8 in the right eye (OD) and 2/8 in the left eye (OS). Slit lamp examination was significant for bilateral conjunctival pallor. Dilated fundoscopic examination revealed Roth spots, macular edema, perivascular cotton-wool spots, extensive intra-retinal and pre-retinal hemorrhages, and chronic subhyaloid hemorrhages bilaterally (Figure 1). Vitreous was clear and optic nerves were sharp, pink, and without evidence of infiltration bilaterally.

Differential diagnoses included infectious, inflammatory, and neoplastic etiologies. A hematologic workup revealed a high white blood cell (WBC) count (425 k/mm^3^) with elevated eosinophils and myelocytes (42%), as well as anemia (hemoglobin 6.2 g/dL, hematocrit 16.8%, mean corpuscular volume (MCV) 112 fL) and thrombocytopenia (15 k/mm^3^). Hematologic markers concerning for tumor lysis syndrome included hypocalcemia (8.2 mg/dL) and elevated lactate dehydrogenase (1063 U/L) while potassium levels were within normal limits (4.0 mEq/L). Infectious workup, blood cultures, fungal workup, and viral panels were negative. Bone marrow biopsy showed markedly increased eosinophils without an increase in blasts, consistent with chronic eosinophilic leukemia (CEL). The aspirate smears revealed a markedly increased eosinophilic component including mature segmented forms and precursors (42%). Immunochemistry showed an atypical myeloid population expressing CD13, CD33, CD11b, CD11c, CD9, and CD38. Flow cytometry was negative for HLA-DR, CD15, CD16, CD64, CD14, and immature markers (CD34, CD117). Polymerase chain reaction (PCR) testing came back negative for BCR-ABL and demonstrated a CHIC2 gene deletion, indicating a favorable prognosis with tyrosine kinase inhibitor therapy. PCR also revealed a PDGFRA gene rearrangement.

**Figure 2:**
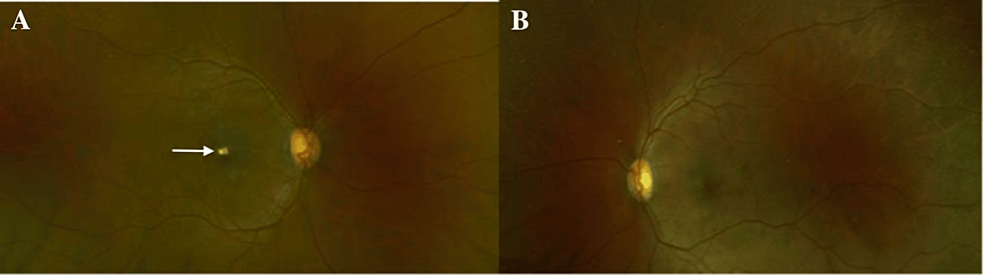
Color fundus photographs after treatment of leukemic retinopathy Color fundus photography at the seven-month follow-up depicting the resolution of hemorrhages in the right (A) and left (B) eyes. There is also the presence of right eye foveal exudate (solid white arrow). There is no evidence of left eye foveal exudate on fundus photography.

**Figure 3:**
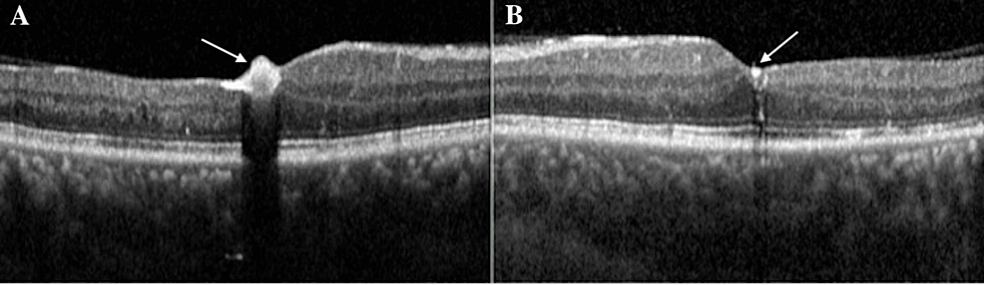
Optical coherence tomography after treatment of leukemic retinopathy Optical coherence tomography (OCT) of the macula at the seven-month follow-up further depicts foveal exudate in the right eye (A, solid white arrow) and minimal residual foveal exudate in the left eye (B, solid white arrow). There is no evidence of intraretinal or subretinal fluid in either eye.

The patient was admitted to the oncology service for treatment with leukapheresis, granulocyte colony-stimulating factor (G-CSF) injections, hydroxyurea, and imatinib, with allopurinol added for tumor lysis syndrome prophylaxis. He was given infection prophylaxis for seven days: acyclovir 400 mg PO BID, fluconazole 200 mg PO once a day, and ciprofloxacin 500 mg PO BID. The inpatient treatment regimen for CEL consisted of the following: two rounds of leukapheresis, several blood and platelet transfusions, one dose of G-CSF, allopurinol (300 mg PO once a day), hydroxyurea (500 mg q12 hours for 4 days), and imatinib (unspecified starting dose lowered to 100 mg PO once a day due to leukopenia). Upon discharge two weeks later, maintenance therapy of daily 100 mg imatinib led to normalized WBC count and resolution of fatigue and dyspnea. BCVA improved to 20/30 OD and 20/25 OS at the seven-month follow-up. Macular edema and retinal hemorrhage resolved after treatment when the patient was seen at his seven-month follow-up (Figures 2, 3), with residual foveal exudate present in the right (Figures 2A, 3A) and left (Figure 3B) eyes.

## Discussion

Leukemia is a systemic hematological disease, with retinal involvement as the most common ocular manifestation [2-4]. Leukemic retinopathy may arise from direct infiltration of cancerous leukocytes. It can also be a consequence of leukemia-induced hematologic abnormalities, which manifest as intraretinal hemorrhages (e.g., dot-blot hemorrhages, flame hemorrhages, Roth spots), preretinal hemorrhages, and cotton-wool spots [5,6]. Besides CEL, leukemic retinopathy has been noted in other leukemias such as acute lymphoblastic leukemia (ALL), acute myeloid leukemia (AML), chronic myeloid leukemia (CML), and adult T-cell leukemia [1]. In this case, leukemic retinopathy was one of the first presenting signs of CEL, which led to our patient receiving a more immediate treatment. Regular follow-up with oncology specialists and ophthalmologists is necessary to monitor the patient’s condition and treatment efficacy.

The presence of ocular involvement in leukemia indicates aggressive systemic disease and portends a poor prognosis [7,8]. Ohkoshi and Tsiaras reported that the five-year survival rate was significantly lower in leukemia patients with leukemic retinopathy on presentation than in those without ophthalmic involvement (21.4% vs. 45.7%) [7]. This is due to a higher likelihood of central nervous system (CNS) involvement in patients exhibiting ophthalmic manifestations of leukemic retinopathy, which is a poor prognostic factor [7]. Abu el-Asrar et al. prospectively evaluated the prognostic importance of retinopathy in adult and pediatric leukemia patients, reporting that the three-month mortality rate of patients with cotton-wool spots is eight times higher than in patients without these retinal lesions. Cotton-wool spots are a product of occluded precapillary arterioles and resultant retinal ischemia, which signify a disease state that is clinically and hematologically active [8]. Therefore, the presence of any retinal hemorrhage or cotton-wool spot in a patient with no apparent systemic cause should prompt physicians to order a complete blood count, including WBC differential, to rule out leukemia and other hematologic irregularities [9].

Once the diagnosis of leukemia has been established, treatment of leukemic retinopathy involves treating the underlying cause with systemic chemotherapy [6]. Imatinib, a BCR-ABL tyrosine kinase inhibitor, has shown promising systemic treatment of different hematological diseases, including myeloproliferative neoplasms with eosinophilia that have evidence of PDGFRA rearrangement. Allopurinol, a xanthine oxidase inhibitor, is often added to imatinib for prophylaxis against tumor lysis syndrome [8]. Treatment regimens with imatinib have not only shown leukemic improvement, but cases have shown resolution of retinal hemorrhages and retinal infiltrates on fundus photography as soon as the one-month follow-up [10,11].

After induction chemotherapy, physicians may consider additional therapies, such as hydroxyurea and leukapheresis, for leukemic treatment. Hydroxyurea given orally at a dose of 50-100 mg/kg daily can reduce the absolute WBC count by 50-80% percent within 48 hours [12]. Leukapheresis, the direct removal of WBCs from circulation, is another adjunct therapy and has been shown to improve VA in patients with retinal involvement [13]. External radiation therapy may be indicated for cases of optic nerve and/or orbital involvement, however, it should be used sparingly due to the risk of radiation-induced retinopathy and cataracts [8].

In most cases, treatment of leukemia by systemic chemotherapy or radiation resolves primary and secondary leukemic retinopathy within the first two months [11,14,15]. If vitreoretinal leukemic infiltration, which can manifest as vitreous cell clumping or yellow-white subretinal infiltrates, persists despite systemic therapy, chemotherapeutic agents, such as methotrexate, can be injected intravitreally [15]. This approach may reduce systemic chemo-drug toxicity when considering additional systemic chemotherapy [8].

Sequelae of untreated leukemic retinopathy include choroidal neovascularization and tractional retinal detachments. In patients with choroidal neovascularization, intravitreal anti-vascular endothelial growth factor (VEGF) agents may be used. If persistent vitreoretinal hemorrhages, vitreomacular traction, or retinal detachments arise in the setting of leukemic retinopathy, a pars plana vitrectomy is indicated [9].

## Conclusions

In cases of unexplained retinal hemorrhages, a high index of suspicion for blood dyscrasias should warrant hematologic evaluation. This patient's visual complaints resulted in a workup that led to the diagnosis of eosinophilic leukemia, with prompt treatment allowing for a favorable prognosis. Ophthalmologists should thus be alert to retinal presentations of leukemia, as a comprehensive eye exam may lead to timely diagnosis and early intervention.
